# Polymicrobial Transjugular Intrahepatic Portosystemic Shunt Infection in the Setting of a Prior Hepaticojejunostomy Anastomosis: A Case Report

**DOI:** 10.1089/crpc.2016.0013

**Published:** 2016-10-01

**Authors:** Prerna Gupta, Timothy R. Donahue

**Affiliations:** ^1^ Department of Surgery, David Geffen School of Medicine at UCLA, Los Angeles, California.; ^2^ Department of Molecular and Medical Pharmacology, David Geffen School of Medicine at UCLA, Los Angeles, California.

**Keywords:** TIPS, hepaticojejunostomy, biliary tree infections, neuroendocrine pancreatic tumor, endotipsitis

## Abstract

**Background:** Vegetative transjugular intrahepatic portosystemic shunt (TIPS) infections are a rare complication of TIPS placement. Cases have been reported in the literature and one study estimated incidence to be 1%.^1^ The vast majority of cases were reported in the setting of cirrhosis. Here, we report a case of vegetative polymicrobial TIPS infection refractory to broad spectrum antibiotics in a patient with a prior hepaticojejunostomy anastomosis as part of a Whipple procedure for a pancreatic neuroendocrine tumor.

**Case Presentation:** A 40-year-old gentleman with pancreatic neuroendocrine tumor underwent neoadjuvant chemoradiation therapy and became eligible for tumor resection. A pancreaticoduodenectomy (Whipple resection) with en bloc superior mesenteric vein (SMV) and portal vein–splenic vein confluence resection was performed. The patient developed SMV stenosis, and a TIPS was placed to access the SMV for stent placement. The patient eventually developed recurrent fevers because of *Escherichia coli* and Enterococcal bacteremia that did not resolve with extended courses of various antibiotics, including meropenem, vancomycin, daptomycin, ertapenem, caspofungin, and piperacillin–tazobactam. The TIPS was eventually removed with an interventional radiology procedure; however, the patient ultimately succumbed to sepsis from antibiotic-resistant bacteria.

**Conclusion:** Here we present a case of endotipsitis in a patient with a biliary enteric anastomosis who did not respond to antibiotic therapy. We caution the use of TIPS in patients with this anatomy, as the biliary tree is inevitably colonized with enteric bacteria and in contact with the intraparenchymal hardware of the TIPS.

## Introduction and Background

Transjugular intrahepatic portosystemic shunts (TIPSs) are now routinely placed in patients with cirrhosis for a variety of indications. It has proven to be an effective treatment for a population of patients who often die from gastrointestinal bleeding.^1^ Typically, these patients are immunosuppressed and it is placed to prevent recurrent bleeding. Complications include incorrect placement, capsule perforation, and infections have been reported but are of the rarer complications.^1^ Here, we report the first case of vegetative TIPS infection in a patient with prior hepaticojejunostomy anastomosis.

## Presentation of the Case

The patient is a 40-year-old male who was diagnosed with a biopsy-proven neuroendocrine tumor that was locally progressed, involving the superior mesenteric vein (SMV) and artery. He underwent downstaging multiagent chemotherapy for over 1 year, during which he developed malabsorption and chronic diarrhea. He became dependent on total parenteral nutrition. However, the tumor shrunk away from the artery and became amenable to resection. A pancreaticoduodenectomy with en bloc SMV and portal vein–splenic vein confluence resection was performed. The splenic vein was ligated and the SMV–portal vein were reconstructed with his saphenous vein. The pathology revealed PNET grade 2 T3N1 disease. He then received additional adjuvant therapy.

Months later, the patient then developed splenomegaly and hypersplenism, recurrent upper GI bleeding, and ascites. He was found to have esophageal and gastric varices that were banded, and he ultimately underwent a splenectomy for the left-sided portal hypertension. The bleeding stopped but the ascites persisted. An SMV venogram was performed, which corroborated with the CT findings that the SMV graft was occluded. After a trial of anticoagulation, he underwent a transhepatic SMV stent placement by interventional radiology (IR). A liver biopsy done at the time revealed metastatic neuroendocrine tumor; the ascites was not malignant.

He then underwent hepatic arterial infusion of Y90 beads. Before that therapy was completed, the ascites reaccumulated, and the SMV stent was again found to be occluded.

Recannulation was attempted by IR through a transjugular intrahepatic approach. The procedure was terminated as the patient went into septic shock from instrumentation of the liver, and he became bacteremic. The blood culture grew *Escherichia coli* and enterococcus faecalis. Approximately 5 days thereafter, the procedure was reattempted. This time, a TIPS was placed in the hepatic parenchyma because of the high likelihood of the need for repeat interventions. Recanalization of the SMV stent was attempted using AngioJet and tPA, leading to brisk flow in the SMV/PV and TIPS. After the procedure, the patient relapsed into septic shock, with blood cultures positive for enterococcus and *E. coli*. The infection was treated with meropenem and vancomycin. He was eventually discharged home after serial negative blood cultures.

The patient re-presented to the hospital soon thereafter with recurrent sepsis. The infection was initially attributed to either a recurrent pleural effusion, hospital-acquired pneumonia, or a peripherally inserted central catheter line infection. He was continued on ertapenem and daptomycin, which were also being administered at home. Ultimately, the TIPS was found to be rethrombosed on imaging, and his blood cultures grew enterococcus faecalis. The thrombosed TIPS was implicated to be the source and he underwent another thrombolysis procedure, which resulted in another episode of septic shock.

**FIG. 1.**
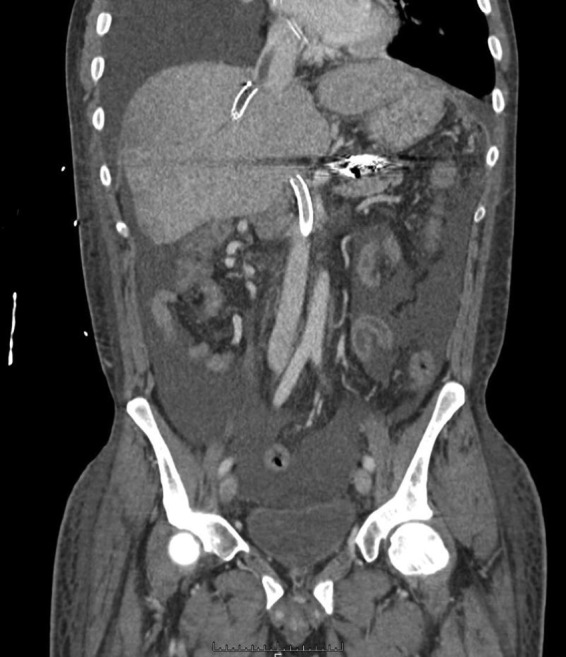
Coronal cut of a CT scan showing the (i) TIPS with the infected thrombus extending to the right atrium, (ii) occluded superior mesenteric vein stent, and (iii) abdominal ascites. TIPS, transjugular intrahepatic portosystemic shunt.

Over the ensuing next few months, the patient continued to return to the hospital with recurrent sepsis, despite being on high dose courses of broad spectrum antibiotics. The TIPS occluded once again, and the thrombus was now found extended to the right atrium (Fig. 1). Using IR techniques, the TIPS was able to be removed; it was infected with yeast. The patient was treated with caspofungin.

Even after the TIPS removal, the patient presented once more with sepsis; blood cultures were positive for Enterobacter. He was transitioned to palliative care, discharged home, and passed away within a week.

## Discussion

Here, we identify a case of persistent sepsis after a TIPS procedure in a patient who had undergone a Whipple resection that included a hepaticojejunostomy. At the time of TIPS placement, the patient had a significant comorbidity burden, including a recently resolved episode of bacteremia and sepsis, and a metastatic neuroendocrine tumor. We believe that these contributed to a recalcitrant infection of the TIPS that needed to be removed, and ultimately contributed to his mortality.

Several long-term complications have been noted in the literature in patients undergoing TIPS with hardware placement. One study identified predictors of mortality. These included a high medical comorbidity burden, hepatitis C virus infection, and low serum albumin.^2^ As previously discussed, our patient had an extensive medical comorbidity burden, along with low serum albumin. Colonization of the biliary tree and its associated outcomes are controversial in the literature.

Vegetative infections of TIPS have also been described. Whereas the etiology of infection in other endotipsitis cases may be less clear,^3^ in this patient who had a hepaticojejunostomy anastomosis, we believe that the TIPS was likely seeded from the colonized biliary tree. One study describes the bacterial pattern often found in stent-associated acute cholangitis to be polymicrobial, because of Enterococci, Enterobacteriaceae, and Candida spp.^4^ This patient, in particular, presented with *E. coli* and Enterobacter sepsis, organisms that may be common in biliary indwelling device infections. In addition, the patient presented here ultimately grew Candida from the TIPS culture. Another study confirmed that biofilms were the major mechanism by which TIPS vegetations became difficult to eradicate.^1^ These findings further support our suspicion that the etiology in this particular patient was likely because of enteral colonization of the biliary tree.

Whereas other patients have responded to antibiotics, ours was refractory to even long-term and broad spectrum drugs. One patient with endotipsitis with blood cultures positive for *Klebsiella oxytoca* and *E. coli* was successfully treated with piperacillin–tazobactam and gentamicin, with slow improvement and eventual transition to ciprofloxacin.^1^ The same study reported that *Staphylococcus aureus* and *Candida* spp. endotipsitis infections are particularly difficult to eradicate, not responding to antimicrobials alone, similar to our patient who eventually grew Candida.^1^ Our patient developed a polymicrobial and fungal infection that was not responsive to all rational drugs administered.

This patient is also unique from other reported cases in that the TIPS thrombus extended into the venocaval junction within the heart. This reflects the degree of infection and also was likely worsened by his elevated systemic inflammatory state caused by the metastatic neuroendocrine tumor, unique from the classically immunocompromised cirrhotic patients who receive TIPSs.

## Conclusion

TIPS infection or “endotipsitis” poses a unique diagnostic and management challenge. Although they often have important treatment benefits, they pose an infection risk that can be fatal. This case illustrates a recalcitrant infection in a patient who had previously undergone a biliary enteric anastomosis. From this experience, we caution TIPS placement in this setting, as the biliary tree becomes colonized with bacteria that are in contact with the shunt in the liver parenchyma.

## Abbreviations Used

CT: computed tomography
IR: interventional radiology
SMV: superior mesenteric vein
TIPS: transjugular intrahepatic portosystemic shunt
